# Metabotropic glutamate receptor 5 (mGluR5) is associated with neurodegeneration and amyloid deposition in Alzheimer’s disease: A [^18^F]PSS232 PET/MRI study

**DOI:** 10.1186/s13195-024-01385-z

**Published:** 2024-01-12

**Authors:** Jie Wang, Yingfang He, Xing Chen, Lin Huang, Junpeng Li, Zhiwen You, Qi Huang, Shuhua Ren, Kun He, Roger Schibli, Linjing Mu, Yihui Guan, Qihao Guo, Jun Zhao, Fang Xie

**Affiliations:** 1 Department of Nuclear Medicine &PET Center, Huashan Hospital, Fudan University, Shanghai, China; 2 Department of Chemistry and Applied Biosciences, Center for Radiopharmaceutical Sciences, Institute of Pharmaceutical Sciences, ETH Zurich, CH-8093 Zurich, Switzerland; 3 Department of Nuclear Medicine, Shanghai East Hospital, School of Medicine, Tongji University, Shanghai, China; 4 Department of Gerontology, Shanghai Jiaotong University Affiliated Sixth People’s Hospital, Shanghai, China

**Keywords:** Metabotropic glutamate receptor 5, Amyloid deposition, Glucose metabolism, Cognitive performance, Plasma biomarkers

## Abstract

**Background:**

Metabotropic glutamate receptor 5 (mGluR5) is involved in regulating integrative brain function and synaptic transmission. Aberrant mGluR5 signaling and relevant synaptic failure play a key role in the initial pathophysiological mechanism of Alzheimer’s disease (AD). The study aims to investigate the association between mGluR5 availability and AD’s biomarkers and cognitive function.

**Methods:**

We examined 35 individuals with mGluR5 tracer [^18^F]PSS232 to assess mGluR5 availability, and with [^18^F]Florbetapir PET to assess global amyloid deposition, and [^18^F]FDG PET to assess glucose metabolism. The plasma neurofilament light (NfL) and p-tau181 levels in a subset of individuals were measured (*n* = 27). The difference in mGluR5 availability between the AD and normal control (NC) groups was explored. The associations of mGluR5 availability with amyloid deposition, glucose metabolism, gray matter volume (GMV), neuropsychological assessment scores, and plasma biomarkers were analyzed.

**Results:**

The mGluR5 availability was significantly reduced in AD patients’ hippocampus and parahippocampal gyrus compared to NCs. Global amyloid deposition was positively associated with mGluR5 availability in the AD group and reversely associated in the NC group. The mGluR5 availability was positively correlated with regional glucose metabolism in the overall and stratified analyses. The availability of mGluR5 in the hippocampus and parahippocampal gyrus demonstrated a strong relationship with the GMV of the medial temporal lobe, plasma p-tau181 or NfL levels, and global cognitive performance.

**Conclusions:**

[^18^F]PSS232 PET can quantify the changes of mGluR5 availability in the progression of AD. mGluR5 availability correlated not only with neuropathological biomarkers of AD but also with neurodegenerative biomarkers and cognitive performance. mGluR5 may be a novel neurodegenerative biomarker, and whether mGluR5 could be a potential therapeutic target for AD needs to be further studied.

**Supplementary Information:**

The online version contains supplementary material available at 10.1186/s13195-024-01385-z.

## Introduction

Alzheimer’s disease (AD) is a progressive neurodegenerative disease and the most common type of dementia, accounting for two-thirds of dementia cases [1]. AD patients suffer noticeable symptoms, such as memory loss and cognitive impairment. As the disease progresses, continuous damage occurs to nerve cells in different regions, eventually leading to a fatal outcome [2]. The neuropathological hallmarks of AD include amyloid-β (Aβ) plaques, neurofibrillary tangles, and neuronal loss [3]. To achieve a biological definition of AD, biomarkers in the cerebrospinal fluid (CSF) or via positron emission tomography (PET) have been recommended for use in clinical observational cohort studies [4].

Metabotropic glutamate receptor 5 (mGluR5) is an excitatory G-protein coupled receptor and predominately localized in the postsynaptic terminals but is also found presynaptically and in the nuclear membrane expressed primarily in postsynaptic terminals of neurons [5, 6], these receptors are involved in the regulation of integrative brain function and synaptic transmission [7]. Glial cells, namely astrocytes and microglia, can also up-regulate the expression of mGluR5 in response to neuroinflammation or trauma, thereby amplifying a potent neuroinflammatory reaction [8–10]. Aberrant mGluR5 signaling and relevant synaptic failure are suggested to play a key role in the initial pathophysiological mechanism of AD [6]. For example, Renner et al. reported that Aβ oligomer-induced abnormal mGluR5 accumulation and overstabilization elevated intracellular calcium levels, leading to synapse deterioration [11]. Moreover, mGluR5 was hypothesized to serve as a coreceptor for cellular-prion-protein-bound Aβ oligomers to activate the tyrosine kinase Fyn, which is closely related to downstream tau phosphorylation [12]. Genetic deletion of mGluR5 reduced the formation of Aβ oligomers that rescued the spatial learning deficits in APPswe/PS1ΔE9 mice [13].

PET using high selectivity and specificity with molecular probes provides opportunities to monitor the alteration of mGluR5 expression levels in physiological and pathological conditions. The non-displaceable binding potential (BP_ND_) of [^18^F]FPEB, a mGluR5-specific radiotracer, revealed significant reductions in the hippocampus mGluR5 availability in sixteen individuals diagnosed with mild cognitive impairment (MCI) due to AD or mild AD dementia [14]. Similarly, Treyer et al. reported that the distribution volume ratios (DVRs) of a carbon-11-labeled mGluR5 tracer ([^11^C]ABP688) were reduced in the bilateral hippocampus and the bilateral amygdala of AD patients compared to normal controls (NCs) [15]. However, to the best of our knowledge, research on the associations of mGluR5 with the neuropathological hallmarks of AD is lacking. Due to the involvement of mGluR5 in AD pathology and its therapeutic potential, it is highly intriguing to investigate the interactions and correlations between various biomarkers in the complex pathogenesis of AD to comprehend the development and advancement of the disease.

In the current study, we sought to explore the associations of mGluR5 with Aβ deposition, glucose metabolism, and gray matter volume in AD patients using non-invasive PET/magnetic resonance imaging (MRI) techniques. A previously reported radioligand, [^18^F]PSS232, was employed for the measurements of mGluR5 expression in human brains [16]. Furthermore, the correlations of mGluR5 availability in the hippocampus and parahippocampal gyrus with plasma biomarkers and cognitive performance were examined.

## Materials and methods

### Participants

Thirty-five participants were recruited in this single-center, cross-sectional study from the memory clinic and communities in Shanghai. All participants underwent a PET/MRI scan with [^18^F]PSS232 to investigate mGluR5 availability and a PET/CT scan with [^18^F]Florbetapir to determine Aβ deposition, including 19 AD and 16 NCs. Fourteen AD patients and sixteen NCs further underwent an [^18^F]FDG PET/CT scan to evaluate glucose metabolism. Blood samples were collected from 13 AD and 14 NCs to measure the plasma concentrations of neurofilament light (NfL) and p-tau181. All the examinations were conducted within a month. Study protocols were approved by the Institutional Review Boards of Fudan University Affiliated Huashan Hospital and Shanghai Jiao Tong University Affiliated Sixth People’s Hospital. Written informed consent was provided by participants or their family members.

The diagnosis of AD was made by experienced neurologists according to the 2018 National Institute on Aging and Alzheimer’s Association (NIA-AA) criteria for probable AD dementia and determined by the positive results of [^18^F]Florbetapir PET scan [4, 17]. Participants who were without amyloid deposition or cognitive impairment were considered NCs. The positive [^18^F]Florbetapir PET images were defined by the method of visual rating according to the guidelines for interpreting amyloid PET [18]. Three board-certified nuclear medicine physicians, including two mid-level and one senior-level title, visually interpreted all [^18^F]Florbetapir PET images independently according to the visual rating guidelines for amyloid PET interpretation, and they are all blinded to the clinical, demographic, and neurological information [19]. The results were determined if more than two physicians made the same judgment, and divided into amyloid PET positive (A+) and negative (A-) groups.

Exclusion criteria are as follows: (1) current major psychiatric diagnoses such as severe depression and anxiety; (2) other neurological conditions which could cause cognitive decline (e.g., cerebrovascular disease, brain tumors, Parkinson’s disease, or epilepsy) rather than AD spectrum disorders; (3) other diseases which could cause cognitive decline (e.g., thyroid dysfunction, severe anemia, syphilis, or HIV); (4) history of psychosis or congenital psychological growth retardation; (5) cognitive decline caused by traumatic brain injury; (6) those who could not complete the study protocol or with contraindications for MRI [19]. Furthermore, participants with a history of smoking were also excluded [20].

### Neuropsychology

All participants received comprehensive neuropsychological assessments, which were revised based on Chinese background [21]. Two global cognitive tests and six neuropsychological tests in three cognitive domains were collected. Auditory verbal learning test (AVLT), 30-minute-long delayed free recall of the AVLT (AVLT-LDR, total scores/12 items) and the AVLT-recognition (total scores/24 items) for memory domain; animal fluency test (AFT, total score) and 30-item Boston naming test (BNT, total score/30 items) for language domain; shape trails test (STT), parts A and B (time to completion) for executive function domain [22, 23] were performed. Mini-Mental State Examination (MMSE) and Montreal Cognitive Assessment-Basic (MoCA-B) as global cognition as well.

### PET and MR imaging

[^18^F]PSS232 PET/MR scans were collected on a 3T PET/MR (uPMR790, United Imaging Healthcare, Shanghai, China). A 30-min static PET/MR scan started at 30-min post-injection of [^18^F]PSS232 (~3.7 MBq/kg body weight), while a 3D Dixon sequence was acquired for attenuation correction and a T1 weighted MR scan was simultaneously acquired using the following parameters: repetition time = 7200 ms, echo time = 3.0 ms, flip angle = 10°, trans axial acquisition matrix = 256 × 329, in-plane resolution = 1 mm × 1 mm, slice thickness = 1 mm, sagittal slice = 176 [24].

Data acquisition of [^18^F]Florbetapir and [^18^F]FDG was conducted using PET/CT scanners (Biograph mCT Flow, Siemens, Erlangen, Germany) with parameters previously described [25, 26]. Twenty-minute scans were conducted at 50 minutes post-injection of ~3.7 MBq/kg (± 10%) of [^18^F]florbetapir intravenously. A 10-minute scan was at 50 min post-injection of ~5.55 MBq/kg (± 10%) of [^18^F]FDG. After the acquisition, the PET images were reconstructed by a filtered back-projection algorithm with corrections for decay, normalization, dead time, photon attenuation, scatter and random coincidences[27].

### Measurements of plasma NfL and p-tau181

The plasma samples collected after the PET studies were stored at -80 °C until further analysis [28]. The plasma biomarkers were measured by the Quanterix Simoa HD-1 platform (changjia). The Neurology 3-Plex A Assay Kit (Lot 502838) and Assay Kit V2 (Lot 502923) were applied for the measurement of NfL and p-tau181, respectively [29]. All the measurements were carried out by technicians blinded to the clinical imaging data. The concentrations of plasma biomarkers are presented in pg/mL.

### Data preprocessing

We used SPM12 (Welcome Trust Centre for Neuroimaging, London, UK; https://www.fil.ion.ucl.ac.uk/spm) for PET image preprocessing following a previously described procedure [26]. Briefly, after reorientation of PET and T1-weighted MR images, PET images were co-registered to the individual T1-weighted images. Then, the T1-weighted images were warped into the standard Montreal Neurological Institute (MNI) space and segmented into gray matter (GM), white matter (WM), and cerebrospinal fluid (CSF); the tissue-labeled images were used for partial volume correction (PVC) of PET images using the Muller-Gartner method [30]. The PET images were also normalized by the transformative parameters from warping the T1-weighted images. Finally, an 8-mm full-width at half-maximum (FWHM) Gaussian kernel was employed to smooth the normalized PET and T1-weighted images.

Quantification was performed by the standardized uptake value ratio (SUVr) using the cerebellum gray matter, pons, and whole cerebellum as reference regions for [^18^F]Florbetapir, [^18^F]PSS232, and [^18^F]FDG, respectively. Regions of interest (ROIs) defined by the Automated Anatomical Labeling (AAL) atlas were applied to the PET data [31]. Specific ROIs, including the frontal, lateral parietal, lateral temporal, and occipital lobes, as well as the insula, amygdala, hippocampus, parahippocampal gyrus, posterior cingulate cortex, anterior cingulate cortex, putamen, precuneus, thalamus, and entorhinal cortex, were used for [^18^F]PSS232 and [^18^F]FDG analysis. Gray matter volume (GMV) was corrected individually by the total intracranial volume, and the hippocampal volume/intracranial volume ratio (HpVr) was calculated.

### Statistical analysis

Voxel-wise analysis was conducted in SPM12, and demographic characteristics and ROI-based analysis were conducted in SPSS 23.0. The group differences in demographic characteristics and neuropsychological scores were compared using the chi-square test for categorical variables and the *t*-test and nonparametric test for continuous variables. ROI-based and voxel-wise comparisons of mGluR5 availability, amyloid deposition, and glucose metabolism between the AD and NC groups were performed using two-sample *t*-tests. Correlations of regional mGluR5 availability with glucose metabolism and GMV were analyzed by a multivariate linear regression model (MLR) based on voxel-wise analyses. The Correlations of regional mGluR5 availability with corresponding regions’ glucose metabolism were used in partial correlation analysis. A general linear regression based on ROI analyses analyzed the correlations of [18F]PSS232 in the hippocampus and parahippocampal gyrus with[18F]FDG SUVr in regions of interest.

The correlations of global amyloid deposition and plasma biomarkers with mGluR5 availability in the hippocampus and parahippocampal gyrus were assessed with partial correlation analysis. The associations of neuropsychological assessments with the hippocampus and parahippocampal gyrus SUVr of [^18^F]PSS232, hippocampus [^18^F]FDG SUVr, and HpVr were assessed with partial correlation analysis, too. For all of the correlation analyses with age, gender and years of education as covariates, we used multiple regression with all covariates to calculate the unstandardized residuals for continuous variables. For voxel-wise analyses, the significance level was set at *p* < 0.05 with peak-level false discovery rate (FDR) correction, and the cluster-defining voxel threshold was set to the default 0.001. Because this is an exploratory study with a small sample size, for other analyses, *p* < 0.05 was considered as a significant difference (two-sided), not corrected for multiple comparisons.

## Results

### Demographic characteristics and clinical assessments

The study sample consisted of 35 participants, including 19 diagnosed with AD and 16 NCs. Table 1 presents the demographic and clinical characteristics of all subjects. Compared with participants in the AD group, the NCs received significantly more years of education (11.81 ± 2.73 vs. 9.72 ± 2.78 years, *p* = 0.035), with less *APOE* ε4 carriers identified (19% vs. 58%, *p* = 0.021). Meanwhile, AD patients showed lower scores in the MMSE (16.44 ± 3.11 vs. 27.88 ± 1.67, *p* < 0.001) and MoCA-B (12.94 ± 3.59 vs. 24.94 ± 2.27, *p* < 0.001) scores.

**Table 1:** Demographic and clinical characteristics of the study cohort

	AD group	NC group	*p* value
Number	19	16	
Percent female	53%	63%	0.833^a^
Age (years)	69.11 ± 7.237	66.00 ± 4.775	0.157^b^
Education (years)	9.72 ± 2.78	11.81 ± 2.73	**0.035**^b^
APOE ε4 carriers	11/19(58%)	3/16(19%)	**0.021** ^a^
MMSE score	16.44 ± 3.11	27.88 ± 1.67	**< 0.001**^b^
MoCA-B score	12.94 ± 3.59	24.94 ± 2.27	**< 0.001**^b^
Global [^18^F]florbetapir SUVr	1.5008 ± 0.3914	1.1941 ± 0.0445	**0.003**^b^
Global [^18^F]FDG SUVr	1.0772 ± 0.0875	1.1740 ± 0.1205	**0.019**^b^
HpVr	0.0034 ± 0.0006	0.0043 ± 0.0004	**< 0.001**^b^
GMV	0.3963 ± 0.0256	0.4224 ± 0.0205	**0.002**^b^

^a^Chi-square test and

^b^independent *t*-test. Values are means ± standard deviations (SDs) unless otherwise noted. Bold values are statistically significant (*p* < 0.05)

*Abbreviations*: *HpVr* the hippocampal volume/intracranial volume ratio, *GMV* Gray matter volume

### Group difference in mGluR5 availability, amyloid deposition, glucose metabolism and gray matter volume

**Fig. 1:**
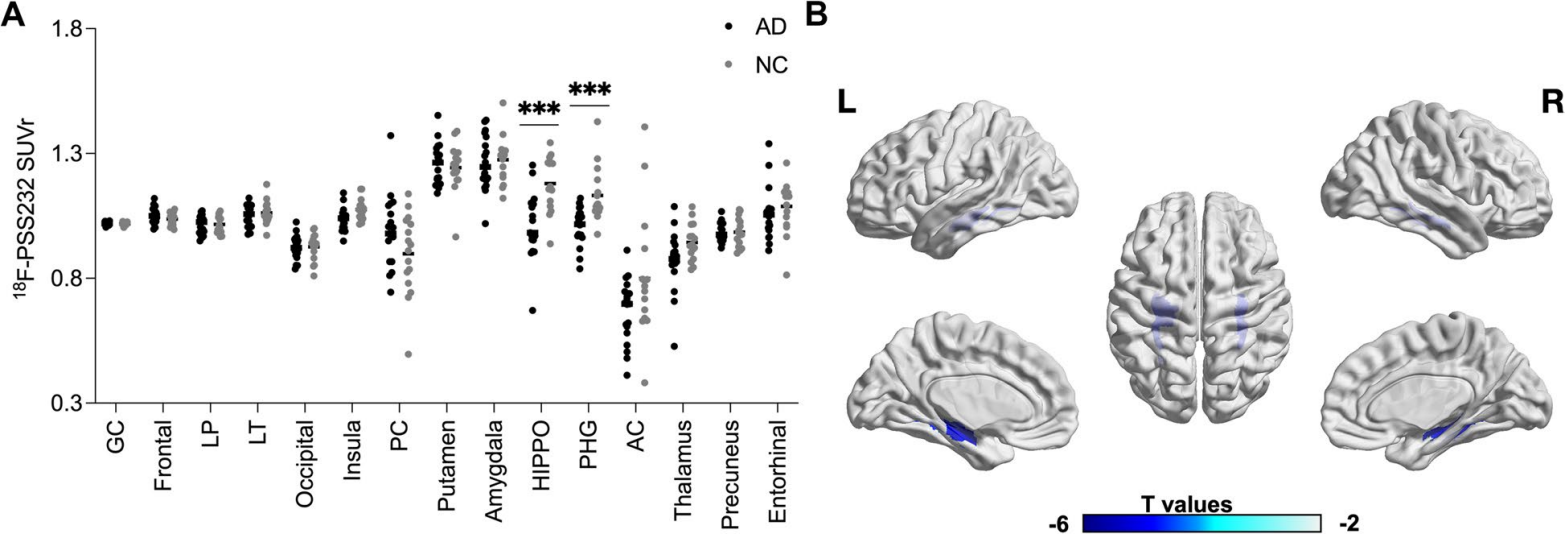
The difference between NC and AD patients in mGluR5 availability using ROI analysis (**A**) and voxel-wise analysis (**B**). The color bar in the voxel-wise analysis represents the T value of the differences between groups with a statistical threshold of *p* < 0.05 with a peak-level FDR correction; the analyses were adjusted for age, gender, and years of education. The group differences in ROI analysis were analyzed using independent *t*-tests with age, education years, and gender as covariates. *** indicates the existence of a significant difference between the two groups with *p* < 0.001, not corrected for multiple comparisons. Abbreviations: GC, global cortex; LP, lateral parietal lobe; LT, lateral temporal lobe; HIPPO, hippocampus; PHG, parahippocampal gyrus. PC, posterior cingulate cortex; AC, anterior cingulate cortex

The differences between NC and AD patients in mGluR5 availability using voxel-wise analysis and ROI analysis were presented in Fig. 1. Using the pons as the reference region in ROI analysis, we found significantly reduced mGluR5 availability in the hippocampus (SUVr: 1.18 ± 0.11 vs. 1.01 ± 0.13, *p* < 0.001) and parahippocampal gyrus (SUVr: 1.13 ± 0.11 vs. 1.01 ± 0.08, *p* < 0.001) in AD patients (Fig. 1A). A similar reduction of mGluR5 availability was observed in the bilateral hippocampus and parahippocampal gyrus in voxel-wise analysis with PVC (Fig. 1B) and without PVC (Supplemental Fig. 1A). While using the cerebellum as the reference region in voxel-wise analysis, we found significantly reduced mGluR5 availability only in partly hippocampus in AD patients, as shown in supplemental Fig. 1B, and there were no differences in PSS232 SUVr between AD and NC based on ROI analysis.

The global amyloid deposition (SUVr: 1.54 ± 0.37 vs. 1.15 ± 0.10, *p* < 0.001) and global glucose metabolism (SUVr: 1.07 ± 0.09 vs. 1.17 ± 0.12, *p* = 0.019) were significantly different between the AD and NC groups according to ROI-based and voxel-wise analyses, as shown in supplemental Fig. 1C and D. A smaller hippocampal volume (0.0034 ± 0.0006 vs. 0.0043 ± 0.0004, *p* < 0.001) and GMV (0.3963 ± 0.0256 vs. 0.4224 ± 0.0205, *p* = 0.002) was found in AD patients compared to those of NC participants. Representative PET images with [^18^F]Florbetapir, [^18^F]FDG and [^18^F]PSS232 in NC and AD participants are shown in supplemental Fig. 2.

### Associations of global amyloid deposition with regional mGluR5 availability

**Fig. 2:**
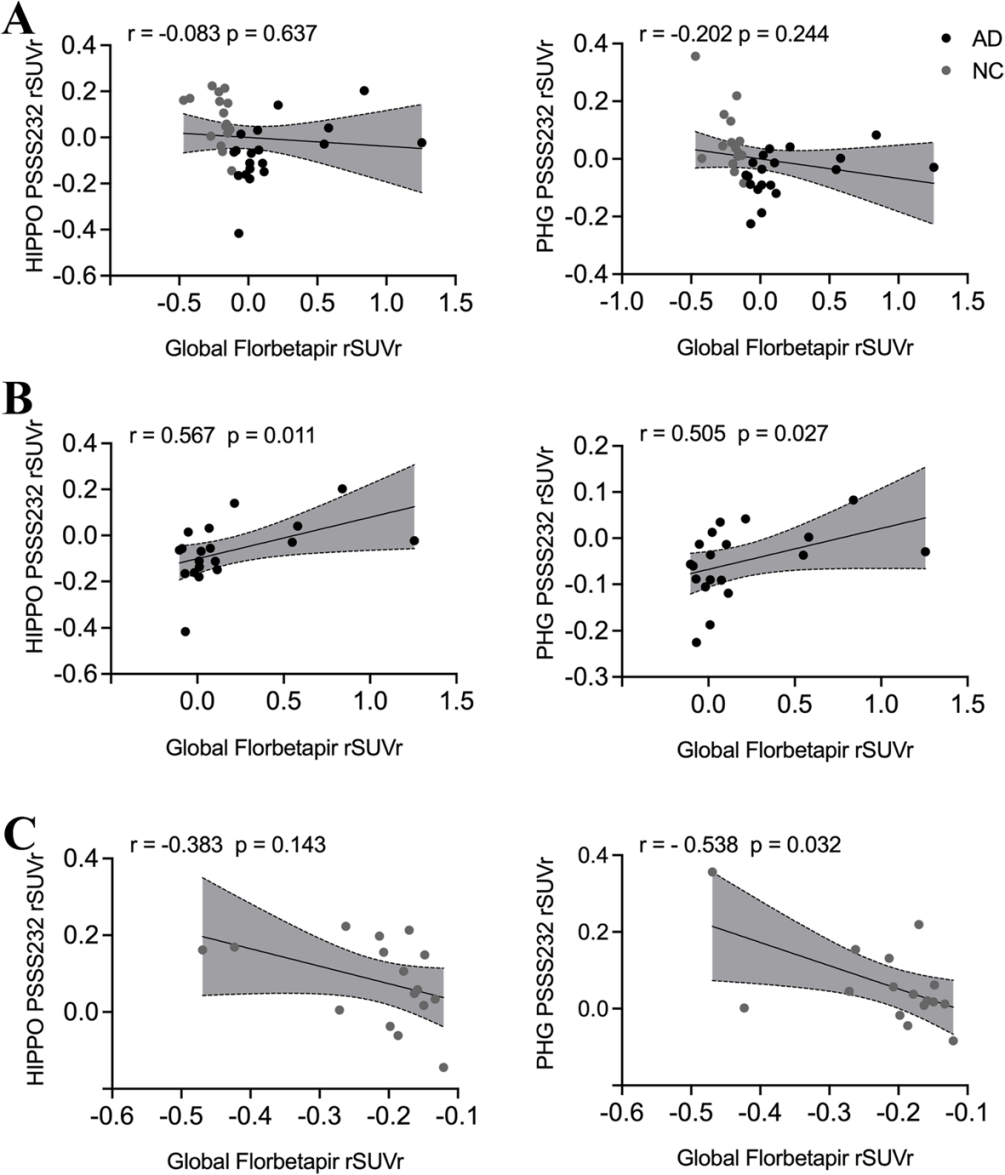
The associations of global amyloid deposition with the regional [^18^F]PSS232 SUVr in the hippocampus and parahippocampal gyrus based on ROI analysis. **A** In the whole cohort, there was no correlation between global amyloid deposition and mGluR5 availability in the hippocampus or parahippocampal gyrus. **B** In the AD group, global amyloid deposition was positively associated with the regional [^18^F]PSS232 SUVr in the hippocampus and parahippocampal gyrus. **C** In the NC group, global amyloid deposition was negatively associated with the regional [^18^F]PSS232 SUVr in the parahippocampal gyrus but not in the hippocampus. The statistical model is partial correlation, with age, education years, and gender as covariates. The dashed lines represent the 95% confidence intervals of the best-fit lines. Abbreviations: HIPPO: hippocampus, PHG: parahippocampal gyrus, rSUVr: the residual of SUVr

Based on ROI analysis, the association of global amyloid deposition with regional mGluR5 availability was further investigated. There was no correlation between global amyloid deposition and mGluR5 availability in the hippocampus or parahippocampal gyrus in the overall cohort (Fig. 2A). In the stratified analysis, global cortex [^18^F]Florbetapir SUVr was positively associated with the regional [^18^F]PSS232 SUVr in the hippocampus (*r* = 0.567, *p* = 0.011) and parahippocampal gyrus (*r* = 0.505, *p* = 0.027) in the AD group (Fig. 2B). However, global cortex [^18^F]Florbetapir SUVr was negatively associated with parahippocampal gyrus [^18^F]PSS232 SUVr in the NC group (*r* = -0.538, *p* = 0.032), as shown in Fig. 2C.

### Associations of regional mGluR5 availability with neurodegenerative biomarkers

**Fig. 3:**
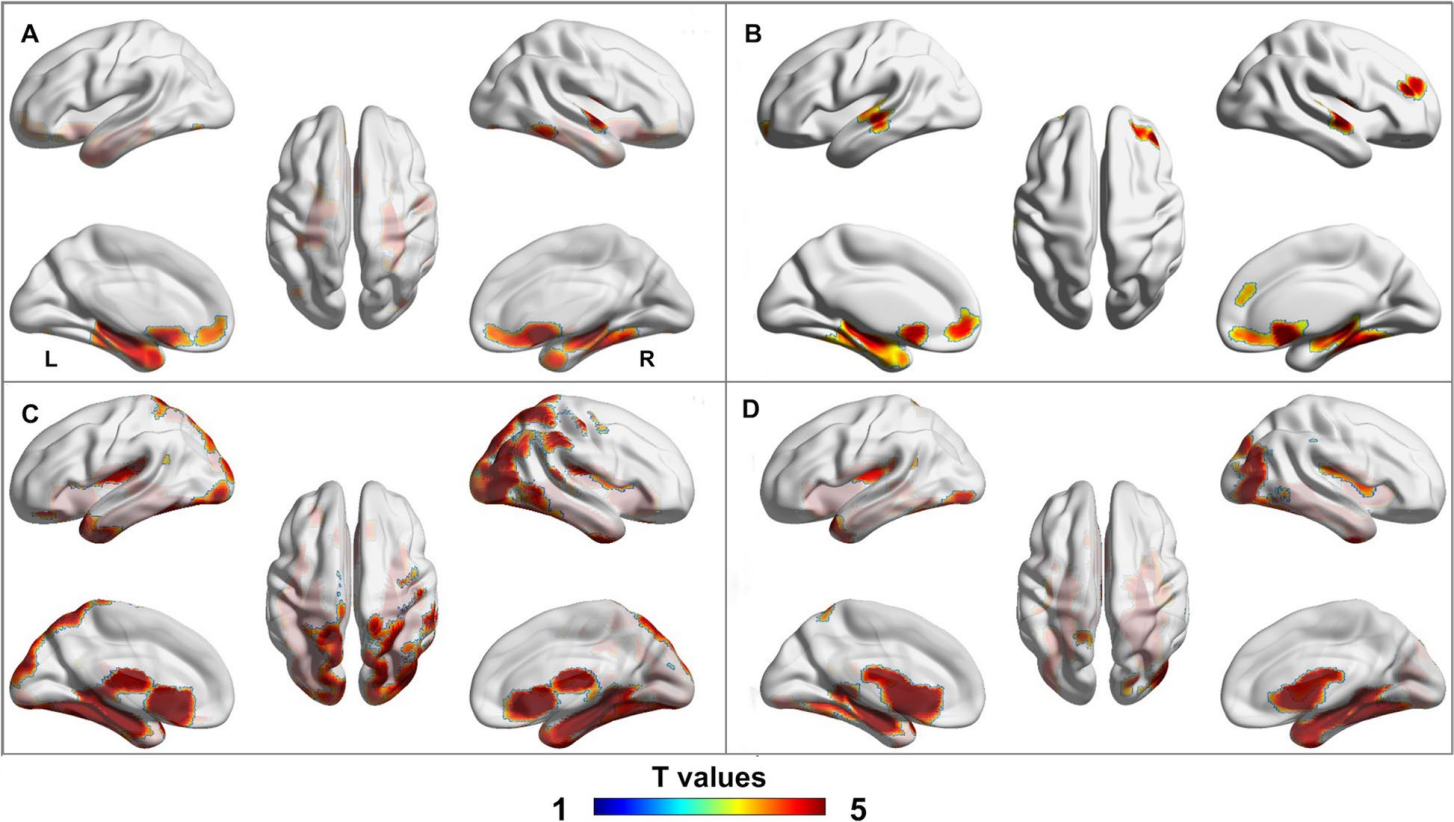
The associations of regional mGluR5 binding with GMV and glucose metabolism by voxel-wise analyses. **A** In the whole cohort, the hippocampus [^18^F]PSS232 SUVr was positively associated with GMV in the bilateral hippocampus, parahippocampal gyrus, and right lateral temporal lobe. **B** In the whole cohort, the parahippocampal gyrus [^18^F]PSS232 SUVr was positively associated with GMV in the bilateral hippocampus, parahippocampal gyrus, right lateral temporal lobe, and anterior cingulate cortex. **C** In the whole cohort, the hippocampus [^18^F]PSS232 SUVr was positively associated with glucose metabolism in the bilateral hippocampus; parahippocampal gyrus, insula occipital, and lateral temporal lobes. **D** In the whole cohort, the parahippocampal gyrus [^18^F]PSS232 SUVr was positively associated with glucose metabolism in the bilateral hippocampus, parahippocampal gyrus, insula, left lateral temporal, and right occipital lobe. The color bar represents the T value with a statistical threshold of *p* < 0.05, peak-level FDR correction

The association of mGluR5 availability in the hippocampus and parahippocampal gyrus with neurodegenerative biomarkers was investigated using voxel-wise analysis. In the overall cohort, the SUVr of [^18^F]PSS232 in the hippocampus and parahippocampal gyrus were positively correlated with GMV in the bilateral hippocampus, parahippocampal gyrus, and right lateral temporal lobe, as shown in Fig. 3A and B. In the stratified analysis, we found no correlation between regional mGluR5 availability and GMV in the AD or NC groups. However, the hippocampus [^18^F]PSS232 SUVr in the overall cohort was positively associated with glucose metabolism in the bilateral hippocampus, parahippocampal gyrus, insula, occipital, and lateral temporal lobe, as shown in Fig. 3C. A similar pattern was observed in the association between the parahippocampal gyrus [^18^F]PSS232 SUVr and glucose metabolism (Fig. 3D). However, this association was observed in similar but smaller regions in the NC group, and in the AD group, this association survived only in the bilateral hippocampus, right parahippocampal gyrus, and superior occipital lobe, as shown in supplemental Fig. 3.

We further performed general linear regression to analyze the association of mGluR5 availability with glucose metabolism using ROI analysis. As shown in Table 2, hippocampus mGluR5 availability was associated with glucose metabolism in the lateral parietal lobe (*p* < 0.001), lateral temporal lobe (*p* = 0.043), occipital lobe (*p* < 0.001), insula (*p* = 0.009), putamen (*p* = 0.039), amygdala (*p* = 0.016), hippocampus (*p* < 0.001), parahippocampal gyrus (*p* < 0.001), anterior cingulate cortex (*p* = 0.003), thalamus (*p* < 0.001), and precuneus (*p* = 0.001) in the overall cohort. A similar tendency was observed in the NC group. However, this effect was observed only in the hippocampus (*p* = 0.034) and thalamus (*p* < 0.001) in the AD group. The hippocampus [^18^F]PSS232 SUVr was associated with global [^18^F]FDG SUVr in the overall cohort (B = 0.375, SE = 0.128, *p* = 0.007) and NC group (B = 0.565, SE = 0.253, *p* = 0.043). Similarly, the parahippocampal gyrus [^18^F]PSS232 SUVr was associated with global and regional [^18^F]FDG SUVr, as shown in supplemental Table 1.

**Table 2:** Effect of the hippocampus [^18^F]PSS232 SUVr on the global and regional [^18^F]FDG SUVr according to general linear regression analyses

	NCs		AD patients		Whole cohort	
	B, β (SE)	*p* value	B, β (SE)	*p* value	B, β (SE)	*p* value
Global cortex	0.565, 0.485 (0.128)	**0.043**	0.046, 0.066 (0.202)	0.824	0.375, 0.485 (0.128)	**0.007**
Frontal lobe	0.501, 0.413 (0.295)	0.111	-0.153, -0.181(0.241)	0.537	0.259, 0.308 (0.151)	0.098
Lateral parietal lobe	0.925, 0.659 (0.282)	**0.005**	0.342, 0.322 (0.291)	0.301	0.686, 0.635 (0.158)	**< 0.001**
Lateral temporal lobe	0.383, 0.454 (0.200)	0.077	-0.042, -0.057 (0.209)	0.793	0.241, 0.371 (0.114)	**0.043**
Occipital lobe	0.619, 0.571 (0.238)	**0.021**	0.351,0.480 (0.185)	0.083	0.520, 0.653 (0.114)	**< 0.001**
Insula	0.805, 0.602 (0.285)	**0.014**	-0.040, -0.077 (0.150)	0.793	0.372, 0.466 (0.134)	**0.009**
Posterior cingulate cortex	-0.387, -0.172 (0.593)	0.525	-0.714, -0.489 (0.367)	0.076	-0.087, -0.058 (0.284)	0.762
Putamen	0.340, 0.336 (0.255)	0.203	-0.023, -0.039 (0.165)	0.893	0.257, 0.379 (0.119)	**0.039**
Amygdala	0.674, 0.619 (0.229)	**0.011**	-0.183, -0.313 (0.160)	0.276	0.323, 0.437 (0.126)	**0.016**
Hippocampus	0.934, 0.861 (0.147)	**< 0.001**	0.324, 0.569 (0.135)	**0.034**	0.644, 0.806 (0.089)	**< 0.001**
Parahippocampal gyrus	0.628, 0.727 (0.158)	**0.001**	0.150, 0.278 (0.149)	0.336	0.405, 0.646 (0.090)	**< 0.001**
Anterior cingulate cortex	1.049, 0.586 (0.388)	**0.017**	0.538, 0.458 (0.302)	0.100	0.627, 0.530 (0.189)	**0.003**
Thalamus	0.780, 0.695 (0.216)	**0.003**	1.012, 0.883 (0.155)	**0.001**	0.823, 0.834 (0.103)	**< 0.001**
Precuneus	1.109, 0.612 (0.383)	**0.012**	0.255, 0.223 (0.322)	0.444	0.774, 0.590 (0.200)	**0.001**
Entorhinal cortex	0.039, 0.066 (0.160)	0.810	-0.165, -0.260 (0.177)	0.369	0.085, 0.163 (0.098)	0.391

The general linear model was used to show [^18^F]PSS232 SUVr in the hippocampus to predict [^18^F]FDG SUVr in each region of interest, every cell represents a separate model

*Abbreviations*: B Unstandardized regression coefficient, *β* Standardized regression coefficient, *SE* Standard error

All analyses were adjusted for age, gender, and years of education. Bold values are statistically significant (*p* < 0.05)

**Fig. 4:**
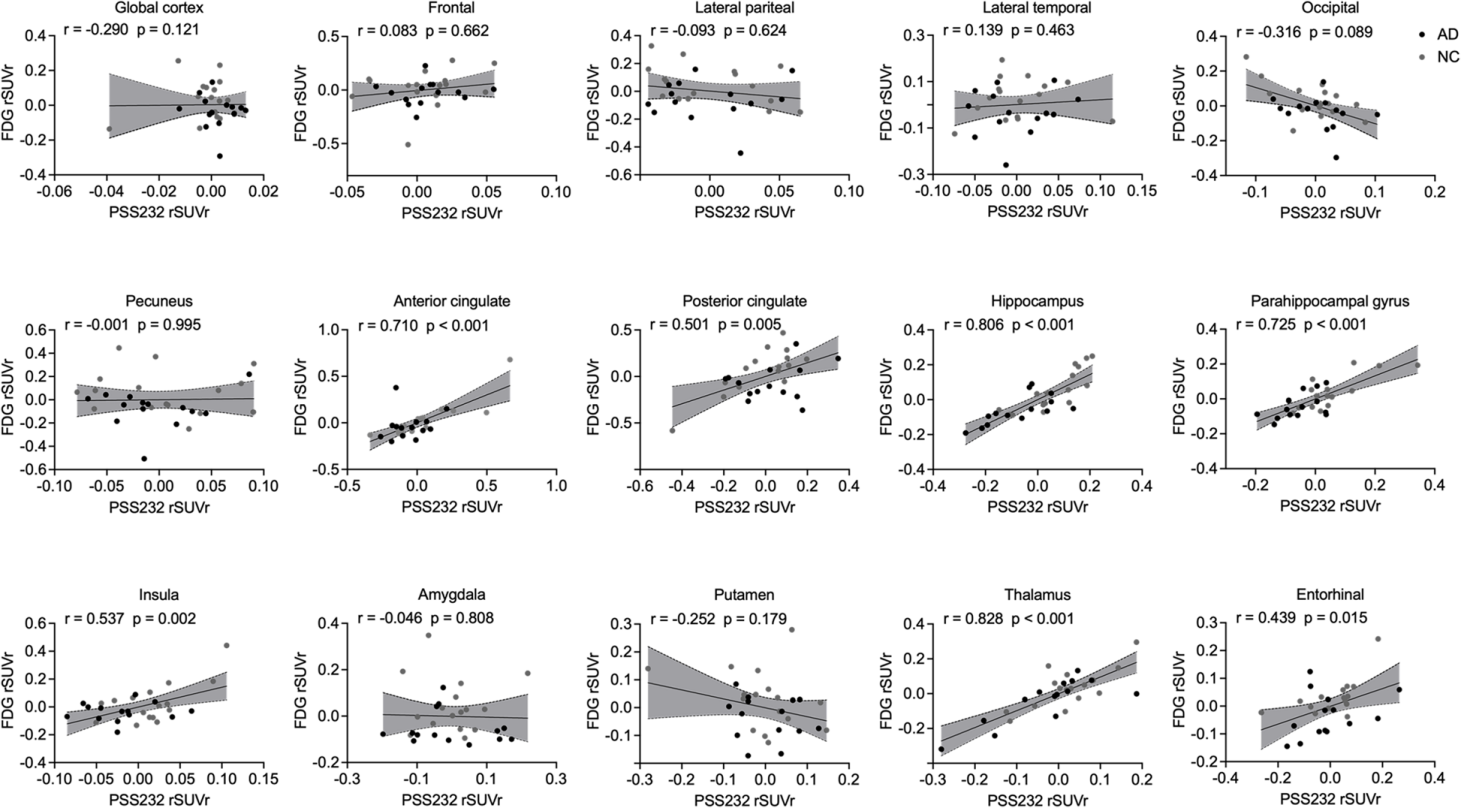
The associations between regional [^18^F]PSS232 SUVr and regional [^18^F]FDG SUVr in the whole cohort. The regional [^18^F]PSS232 SUVr was positively associated with regional glucose metabolism in the posterior cingulate cortex, anterior cingulate cortex, hippocampus, parahippocampal gyrus, insula, thalamus, and entorhinal cortex. The statistical model is partial correlation, with age, years of education, and gender as covariates. The dashed lines represent the 95% confidence intervals of the best-fit lines. Abbreviations: rSUVr: the residual of SUVr

The association between the regional mGluR5 availability and the corresponding region’s glucose metabolism was also investigated. The regional [^18^F]PSS232 SUVr was positively associated with regional [^18^F]FDG SUVr in the posterior cingulate cortex (*r* = 0.501, *p* < 0.001), anterior cingulate cortex (*r* = 0.710, *p* < 0.001), hippocampus (*r* = 0.806, *p* < 0.001), parahippocampal gyrus (*r *= 0.725, *p* < 0.001), insula (*r* = 0.537, *p* = 0.002), thalamus (*r* = 0.828, *p* < 0.001) and entorhinal cortex (*r* = 0.537, *p* = 0.015) in the overall cohort, as shown in Fig. 4. A similar pattern was observed in the stratified analysis in the NC group, but the associations only survived in the hippocampus (*r* = 0.594, *p* = 0.034) and thalamus (*r* = 0.869, *p* < 0.001) in the AD group, as shown in supplemental Figs. 4 and 5.

### Associations between mGluR5 availability and neuropsychological assessments

**Fig. 5:**
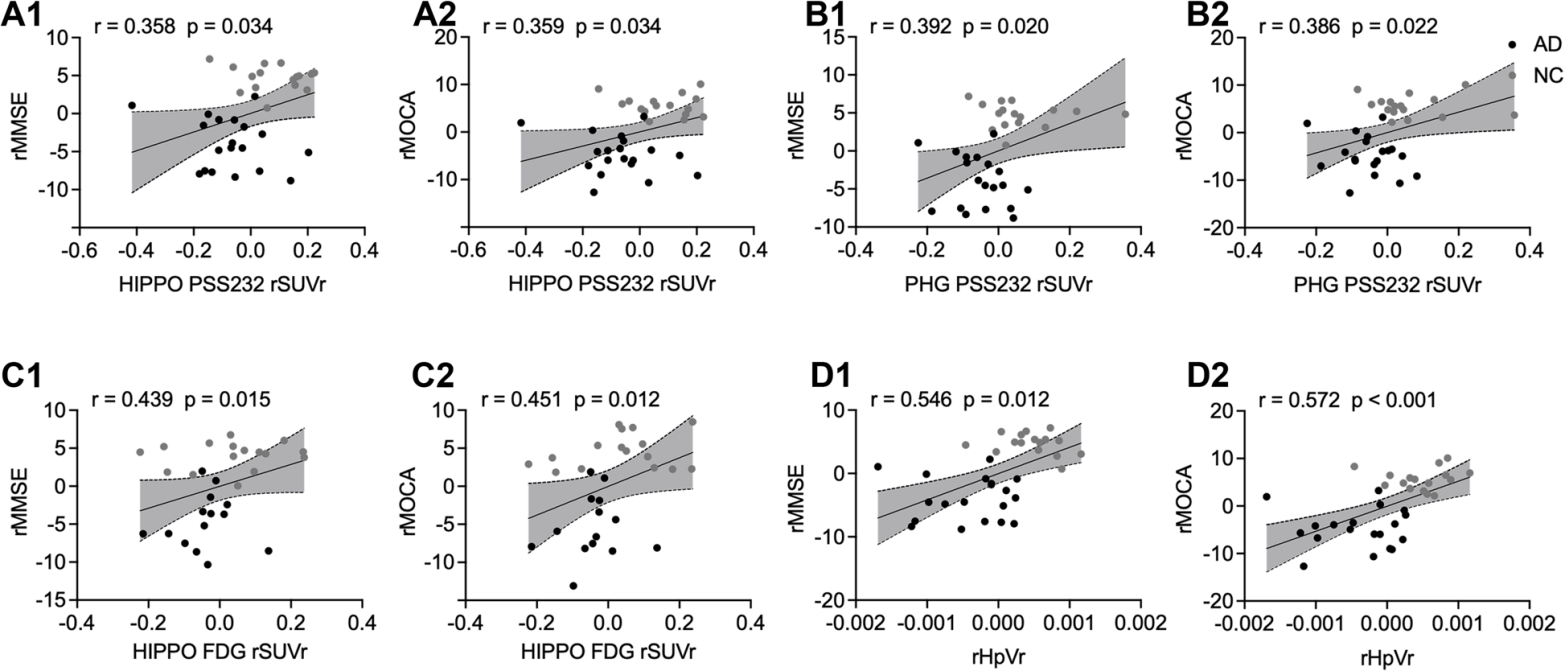
The correlation between cognition scores and image biomarkers in the whole cohort. **A **The [^18^F]PSS232 SUVr in the hippocampus was positively associated with global cognition scores on the MMSE (A1)and MoCA-B (A2). **B** The [^18^F]PSS232 SUVr in the parahippocampal gyrus was positively associated with global cognition scores on the MMSE (B1) and MoCA-B (B2). **C** Hippocampal glucose metabolism was positively associated with MMSE scores (C1) and MoCA-B scores (C2). **D** The HpVr was positively associated with MMSE scores (D1) and MoCA-B scores (D2). The statistical model is partial correlation, with age, years of education, and gender as covariates. The dashed lines represent the 95% confidence intervals of the best-fit lines. Abbreviations: HIPPO: hippocampus, PHG: parahippocampal gyrus, rSUVr: the residual of SUVr, rMMSE: the residual of MMSE, rMoCA-B: the residual of MoCA-B

In the overall cohort, the [^18^F]PSS232 SUVr in the hippocampus and parahippocampal gyrus was positively associated with global cognition scores on the MMSE (*r* = 0.358, *p* = 0.034 vs. *r* = 0.392, *p* = 0.020) and MoCA-B (*r* = 0.359, *p* = 0.034 vs. *r* = 0.386, *p* = 0.022). Hippocampal glucose metabolism and the HpVr were positively associated with MMSE (*r* = 0.439, *p* = 0.015 vs. *r* = 0.546, *p* = 0.001) and MoCA-B (*r* = 0.451, *p* = 0.012 vs. *r* = 0.572, *p* < 0.001) scores in the overall cohort, as shown in Fig. 5. Meanwhile, these associations were absent in the NC and AD groups in the stratified analysis.

We also further analyzed the correlation of mGluR5 availability with memory, language, and executive functioning. In the whole cohort the [^18^F]PSS232 SUVr in the hippocampus and parahippocampal gyrus was positively associated with memory functioning scores on the AVLT -LDR (*r* = 0.526, *p* = 0.034 vs. *r* = 0.509, *p* = 0.042), and it was also positively associated with language functioning scores on the AFT (*r* = 0.496, *p* = 0.029 vs. *r* = 0.457, *p* = 0.036) and BNT (*r* = 0.361, *p* = 0.026 vs. *r* = 0.399, *p* = 0.046). However, there was no correlation between executive functioning and mGluR5 availability in the hippocampus or parahippocampal gyrus in the overall cohort, as shown in supplemental Table 2. Moreover, these associations were not present in the NC and AD groups in the stratified analysis.

### Associations between the regional [^18^F]PSS232 SUVr and plasma biomarkers

**Fig. 6:**
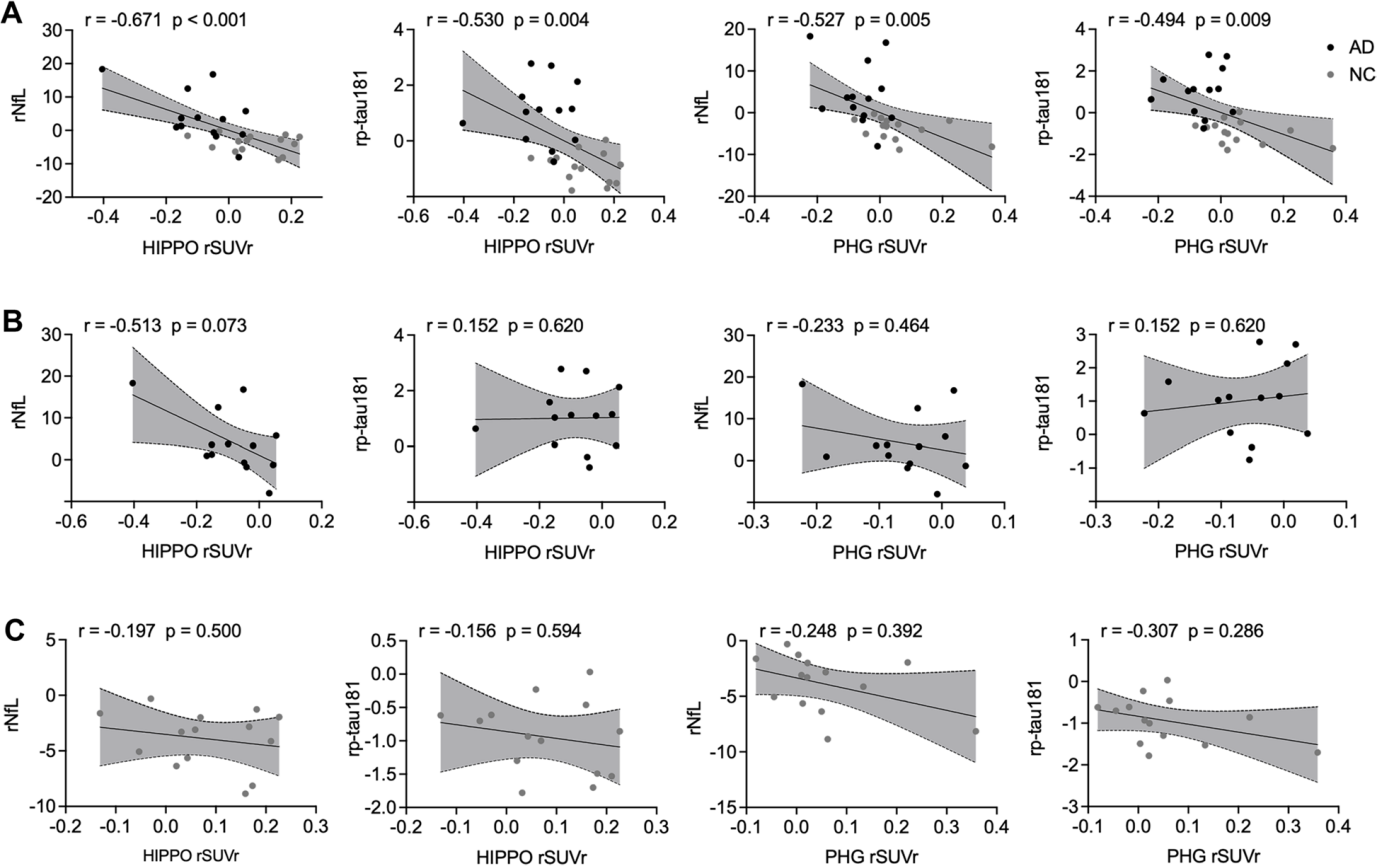
The correlation between mGluR5 availability and plasma biomarkers in the whole cohort. **A** In the whole cohort, (**B**) In the AD group, (**C**) In the NC group. The statistical model is partial correlation, with age, years of education, and gender as covariates. The dashed lines represent the 95% confidence intervals of the best-fit lines. HIPPO: hippocampus, PHG: parahippocampal gyrus. rp-tau 181: the residual of p-tau 181, rNfL: the residual of NfL

We further investigated the associations of mGluR5 availability in the hippocampus and parahippocampal gyrus with the plasma biomarkers NfL and p-tau181, as shown in Fig. 6. The [^18^F]PSS232 SUVr of the hippocampus and parahippocampal gyrus was negatively associated with NfL levels (*r* = -0.671, *p* < 0.001 vs. *r* = -0.527, *p* = 0.005) and p-tau181 levels (*r* = -0.530, *p* = 0.004 vs. *r* = -0.494, *p* = 0.009). However, these associations were absent in the NC and AD groups in the stratified analysis.

## Discussion

mGluR5 has been hypothesized to mediate Aβ oligomer toxicity and to link with amyloid and tau pathology in AD [11]. However, the association of mGluR5 PET biomarkers with key AD “A/T/N” (amyloid/tau/neurodegeneration) biomarkers remains unknown. Using [^18^F]PSS232 PET as a specific-mGluR5 visualization tool, our current work demonstrated a systematic study to investigate the correlations of mGluR5 availability with global amyloid deposition, glucose metabolism, and neurodegenerative biomarkers. The results revealed significantly reduced mGluR5 expression levels in AD patients compared to NCs. In addition, our stratified analysis showed that amyloid deposition was associated with mGluR5 availability in AD and NC groups, respectively. Moreover, mGluR5 availability was closely associated with neurodegenerative biomarkers, cognitive function, and plasma biomarkers, suggesting that mGluR5 could be a novel neurodegenerative biomarker. Whether mGluR5 could be a potential therapeutic target for AD deserves further investigation.

Our findings confirm that mGluR5 availability in the medial temporal lobe is significantly reduced in AD patients. This result is in line with previous reports using [^11^C]ABP688 or [^18^F]FPEB as molecular probes [14, 15]. Notably, instead of the cerebellum suggested as a reference tissue in the first-in-man study [16], pons was used as a reference region in our studies for the quantification of [^18^F]PSS232 signals. This is due to the upregulation of mGluR5 expression in the cerebellum during neuroinflammation of AD [7, 32], which makes it inappropriate as a reference region. The radioactive accumulation of the cerebellum in AD is larger than in NCs, while pons exhibiting highly similar radioactive accumulation in AD and NC were then selected as a reference tissue in our case. The mGluR5 availability was lower in the hippocampus and parahippocampus gyrus in AD patients compared with NCs, both based on voxel-wise and ROI analyses. Therefore, we then focus on these areas for further exploration.

Growing evidence suggests an association between amyloid pathology and mGluR5 in AD. In the early stage of AD, the mGluR5 receptor mediates synaptic toxicity signaling through cellular prion protein (PrPc)/Aβ oligomeric complexes [9]. This aberrant mGluR5 signaling was suggested to inhibit the autophagic clearance of Aβ oligomers and plaques, and consequently led to severe amyloid deposition in patient brains [33]. On the other hand, Aβ-induced neuroinflammation may further induce the upregulation of mGluR5, since mGluR5 is expressed in neurons as well as in glial cells, including activated astrocytes and microglia [8]. In agreement, we observed a positive association of mGluR5 availability in the hippocampus and parahippocampal gyrus with AD patients’ global amyloid deposition. Meanwhile, no and negative associations of global amyloid deposition with mGluR5 availability were found in the overall cohort and NCs, respectively. This finding is consistent with the previous findings by Casley et al., who reported that mGluR5 expression was elevated in astrocytes of AD patients using immunostaining [34]. Considering the protein expression level of mGluR5 fluctuated with the progression of AD in 5xFAD mice mode [35], longitudinal PET imaging of [^18^F]PSS232 on AD patients and healthy volunteers is warranted to investigate whether the alteration of mGluR5 receptors could predict the clinical progression of AD.

Plasma p-tau181 accurately identified healthy elderly controls and individuals with MCI with a positive Aβ-PET scan and also differentiated between individuals with elevated cortical tau deposition [36, 37]. Elevated p-tau181 concentrations correlated with higher Flortaucipir (FTP) -PET uptake and more severe gray matter atrophy in AD-related brain regions [38–40]. In our study, we found plasma p-tau181 was negatively associated with mGluR5 availability both in the hippocampus and parahippocampal gyrus. According to a previous study, Aβ oligomers -mGluR5- PrPc complex could elicit tau hyperphosphorylation by activating the tyrosine kinase Fyn, leading to tau deposition in the brain [41]. So the mGluR5 availability may be closely associated with the “T” biomarkers of AD.

Furthermore, our work demonstrated that mGluR5 availability in the hippocampus and parahippocampal was closely associated with AD-related neurodegenerative biomarkers. We found that the regional mGluR5 availability was positively correlated with the regional glucose metabolism, while the mGluR5 availability in the hippocampus and parahippocampal gyrus was mainly associated with GMV in the medial temporal lobe. The association between mGluR5 availability and glucose metabolism was also validated by a general regression model; specifically, mGluR5 availability in the hippocampus and parahippocampal gyrus predicted global and regional glucose metabolism. These results indicate that mGluR5 is closely associated with glucose metabolism and might become a stronger predictor of glucose metabolism in future studies. Plasma NfL is another neurodegenerative marker that tracks neurodegeneration in AD and is negatively correlated with atrophy, hypometabolism, and cognitive decline [42, 43]. We found that mGluR5 availability in the hippocampus and parahippocampal gyrus was negatively associated with plasma NfL levels. Whether mGluR5 could be an index of glucose metabolism and a novel neurodegenerative biomarker needed to be further explored.

We further investigate the relationship between mGluR5 availability and cognitive function. As expected, AD patients were inferior to the NCs in MMSE and MoCA-B scores. The overall cohort identified a positive association between mGluR5 availability and neuropsychological assessments. These findings could be explained by the physiological role of mGluR5 in the modulation of rapid synaptic transmission and plasticity changes underlying the cognitive processes. It’s worth mentioning that mGluR5-selective negative allosteric modulators were previously reported to reduce the Aβ oligomers and plaques, and to improve the cognitive function in the APPswe/PS1ΔE9 mouse model of AD [44]. Therefore we concluded that pharmacological inhibition of aberrant mGluR5 could potentially work as a novel approach against AD. In which, amyloid reduction monitored by PET imaging served as a positive change on a surrogate endpoint. Considering the intersection of mGluR5 in cognition and amyloid deposition, PET studies using [^18^F]PSS232 might be of great interest to evaluate the clinical outcomes more objectively together with the cognitive subscale test.

There were several limitations in this study. Firstly, PET imaging targeting tau or synaptic density cannot be included in this study. Tau PET can directly reflect the tau pathology in the brain of AD. The synaptic density quantified by synaptic vesicle glycoprotein 2A (SV2A) PET tracer, such as [^11^C]UCB-J [45], needs to be enrolled to further interpret the relationship between the expression level of mGluR5 receptor and integrated local neuronal activity in vivo. Secondly, this is an exploratory study with a small sample size and there was no correction for multiple comparisons at the time of statistical analysis. For those reasons, we need to be careful when drawing far-reaching conclusions from these preliminary findings. Finally, our study was based on cross-sectional, more extensive longitudinal PET studies in a larger cohort are warranted to comprehend the role of mGluR5 expression in AD pathogenesis and cognitive impairment.

## Conclusion

Using a mGluR5-specific molecular probe [^18^F]PSS232 PET could quantify the changes in mGluR5 availability in the progression of AD. mGluR5 availability was positively associated with global amyloid deposition in AD patients, and negatively associated with global amyloid deposition in NCs. mGluR5 availability was also negatively associated with plasma p-tau 181(a “T” biomarker of AD) in the whole cohort. Meanwhile, mGluR5 availability was closely correlated with neurodegenerative biomarkers, such as regional glucose metabolism, hippocampal volume, plasma NfL and global cognitive performance. In total, our findings suggest that mGluR5 might be a novel neurodegenerative biomarker for AD, which provides a new idea for understanding AD-related neurodegenerative diseases. Whether mGluR5 could be a potential therapeutic target for AD needs to be further studied. Further studies, including longitudinal studies, are ongoing to interpret the fluctuation of mGluR5 expression levels during AD progression and its underlying mechanisms.

### Supplementary Information

**Additional file 1:** **Supplemental Table 1.** The effect of parahippocampal gyrus [^18^F]PSS232 SUVr on global and regional [^18^F]FDG SUVr by general linear regression analyses. **Supplemental Table 2.** Associations between mGluR5 availability and neuropsychological assessments. **Supplemental Figure 1.** Group difference of mGluR5 expression, amyloid deposition, and glucose metabolism between NC and AD by voxel-wise analysis. **Supplemental Figure 2.** Representative PET images in the NC and AD patient from [^18^F]Florbetapir (A), [^18^F]FDG (B), and [^18^F]PSS232 (C). **Supplemental Figure 3.** The associations of regional mGluR5 binding with glucose metabolism by voxel-wise analyses in the AD patients and NCs. **Supplemental Figure 4.** The associations between regional [^18^F]PSS232 SUVr and regional [^18^F]FDG SUVr in the NC group. **Supplemental Figure 5.** The associations between regional [^18^F]PSS232 SUVr and regional [^18^F]FDG SUVr in the AD group.

## Data Availability

The data supporting the findings of this study are available on request from the corresponding author. The data are not publicly available due to privacy or ethical restrictions.

## Abbreviations

AD: Alzheimer’s disease
Aβ: Amyloid-β
AAL: Automated Anatomical Labeling
BP_ND_: Non-displaceable binding potential
CSF: Cerebrospinal fluid
DVRs: Distribution volume ratios
FEW: Familywise error
FWHM: Full-width at half-maximum
FDG: Fluorodeoxyglucose
GM: Gray matter
GMV: Gray matter volume
NCs: Normal controls
HIPPO: Hippocampus
HpVr: Hippocampal volume/intracranial volume ratio
mGluR5: Metabotropic glutamate receptor 5
MCI: Mild cognitive impairment
MRI: magnetic resonance imaging
MMSE: Mini-Mental State Examination
MoCA-B: Montreal Cognitive Assessment-Basic
MLR: multivariate linear regression
MNI: Montreal Neurological Institute
NIA-AA: National Institute on Aging and Alzheimer’s Association
NfL: Neurofilament light
PET: Positron emission tomography
PHG: Parahippocampal gyrus
PrPc: Cellular prion protein
PVC: Partial volume correction
ROI: Region of interest
SUVr: Standardized uptake value ratio
rSUVr: The residual of SUVr
WM: White matter
