# Supplementary material for: Metabotropic glutamate receptor 5 (mGluR5) is associated with neurodegeneration and amyloid deposition in Alzheimer’s disease: A [18F]PSS232 PET/MRI study
Source: Alzheimers Res Ther. 2024 Jan 12;16:9. doi: 10.1186/s13195-024-01385-z (PMC10785459; doi:10.1186/s13195-024-01385-z)
Supplement: Supplementary file 1 — Additional file 1: Supplemental Table 1. The effect of parahippocampal gyrus [18F]PSS232 SUVr on global and regional [18F]FDG SUVr by general linear regression analyses. Supplemental Table 2. Associations between mGluR5 availability and neuropsychological assessments. Supplemental Figure 1. Group difference of mGluR5 expression, amyloid deposition, and glucose metabolism between NC and AD by voxel-wise analysis. Supplemental Figure 2. Representative PET images in the NC and AD patient from [18F]Florbetapir (A), [18F]FDG (B), and [18F]PSS232 (C). Supplemental Figure 3. The associations of regional mGluR5 binding with glucose metabolism by voxel-wise analyses in the AD patients and NCs. Supplemental Figure 4. The associations between regional [18F]PSS232 SUVr and regional [18F]FDG SUVr in the NC group. Supplemental Figure 5. The associations between regional [18F]PSS232 SUVr and regional [18F]FDG SUVr in the AD group. [file 13195_2024_1385_MOESM1_ESM.docx]

**Supplemental materials**

**Metabotropic glutamate receptor 5 (mGluR5) is associated with neurodegeneration and amyloid deposition in Alzheimer’s disease: A [^18^F]PSS232 PET/MRI study**

**Supplemental Table 1.** The effect of parahippocampal gyrus [^18^F]PSS232 SUVr on global and regional [^18^F]FDG SUVr by general linear regression analyses.

|  | NCs | | AD patients | | Whole cohort | |
| --- | --- | --- | --- | --- | --- | --- |
|  | B, β, (SE) | *p* value | B, β, (SE) | *p* value | B, β, (SE) | *p* value |
| Global cortical | 0.168, 0.150 (0.295) | 0.578 | -0.354,-0.302(0.323) | 0.295 | 0.249, 0.248 (0.184) | 0.186 |
| Frontal lobe | 0.039, 0.032 (0.433) | 0.907 | -0.703,-0.496(0.356) | 0.072 | 0.081, 0.075 (0.206) | 0.696 |
| Lateral parietal lobe | 0.433, 0.305 (0.361) | 0.250 | -0.045, -0.025 (0.513) | 0.932 | 0.570, 0.406 (0.242) | **0.026** |
| Lateral temporal lobe | 0.132, 0.155 (0.225) | 0.568 | -0.462, -0.382 (0.323) | 0.178 | 0.145, 0.172 (0.157) | 0.363 |
| Occipital | 0.447, 0.407 (0.268) | 0.118 | 0.238, 0.195 (0.346) | 0.504 | 0.530, 0.513 (0.168) | **0.004** |
| Insula | 0.944, 0.698 (0.259) | **0.003** | -0.200, -0.230 (0.245) | 0.429 | 0.586, 0.564 (0.162) | **0.001** |
| Posterior cingulate | -1.051, -0.461 (0.541) | 0.072 | -1.305, -0.535 (0.595) | **0.049** | -0.433, -0.221 (0.361) | 0.241 |
| Putamen | 0.586, 0.573 (0.224) | **0.020** | -0.088,-0.092 (0.274) | 0.754 | 0.453, 0.514 (0.143) | **0.004** |
| Amygdala | 0.922, 0.837 (0.161) | **< 0.001** | -0.143,-0.147 (0.279) | 0.617 | 0.629, 0.654 (0.138) | **< 0.001** |
| Hippocampus | 0.819, 0.747 (0.195) | **0.001** | 0.514, 0.541 (0.231) | **0.046** | 0.812, 0.783 (0.122) | **< 0.001** |
| Parahippocampal gyrus | 0.681, 0.780 (0.146) | **< 0.001** | 0.307, 0.341 (0.244) | 0.233 | 0.591, 0.725 (0.106) | **< 0.001** |
| Anterior cingulate | 1.050, 0.580 (0.394) | **0.019** | 0.813, 0.414 (0.516) | 0.141 | 0.833, 0.542 (0.244) | **0.002** |
| Thalamus | 0.435, 0.383 (0.280) | 0.143 | 1.281, 0.669 (0.411) | **0.009** | 0.782, 0.610 (0.192) | **< 0.001** |
| Precuneus | 0.258, 0.141 (0.485) | 0.602 | -0.299, -0.156 (0.545) | 0.593 | 0.519, 0.305 (0.307) | 0.102 |
| Entorhinal | 0.394, 0.649 (0.123) | **0.006** | -0.377, -0.356 (0.286) | 0.212 | 0.261, 0.383 (0.119) | **0.037** |

Note: The general linear model was used to show [^18^F]PSS232 SUVr in the parahippocampal gyrus to predict [^18^F]FDG SUVr in each region of interest, every cell represents a separate model. Abbreviations: B, unstandardized regression coefﬁcient; β, standardized regression coefﬁcient; SE, standard error. Note: All analyses were adjusted for age, gender, and years of education. Bold values are statistically significant (*p* < 0.05).

**Supplemental Table 2. Associations between mGluR5 availability and neuropsychological assessments**

|  |  | AVLT-LDR | AVLT-recognition | AFT | BNT | STT-A | STT-B |
| --- | --- | --- | --- | --- | --- | --- | --- |
|  |  | r, p value | r, p value | r, p value | r, p value | r, p value | r, p value |
| Whole cohort | Hippocampus | **0.526, 0.034** | 0.276, 0.051 | **0.496,0.029** | **0.361, 0.026** | -0.193, 0.239 | -0.124,0.563 |
|  | Parahippocampal gyrus | **0.509, 0.042** | 0.324, 0.074 | **0.457, 0.036** | **0.399, 0.046** | -0.207, 0.415 | -0.241,0.467 |
| AD group | Hippocampus | -0.447, 0.127 | -0.329, 0.229 | -0.147,0.548 | -0.246, 0.673 | 0.127, 0.889 | 0.113,0.972 |
|  | Parahippocampal gyrus | -0.436, 0.231 | -0.432,0.378 | -0.142, 0.562 | -0.293, 0.542 | 0.237, 0.798 | 0.149, 0.871 |
| NC group | Hippocampus | 0.264,0.323 | 0.336,0.498 | 0.237,0.643 | 0.364,0.397 | -0.264, 0.237 | -0.187,0.253 |
|  | Parahippocampal gyrus | 0.113,0.577 | 0.275, 0.362 | 0.172,0.525 | 0.302,0.129 | -0.322, 0.129 | -0.298, 0.109 |

Note: The partial correlation was used to analyze the associations of [^18^F]PSS232 SUVr in the hippocampus and parahippocampal gyrus with memory (AVLT-LDR and AVLT-recognition), language (AFT and BNT), and executive functioning (STT-A and STT-B), respectively. Note: All analyses were adjusted for age, gender, and years of education. Bold values are statistically significant (*p* < 0.05). Abbreviations: AVLT-LDR, 30-minute-long delayed free recall of the auditory verbal learning test; AFT, animal fluency test; BNT, Boston naming test; STT, shape trails test.


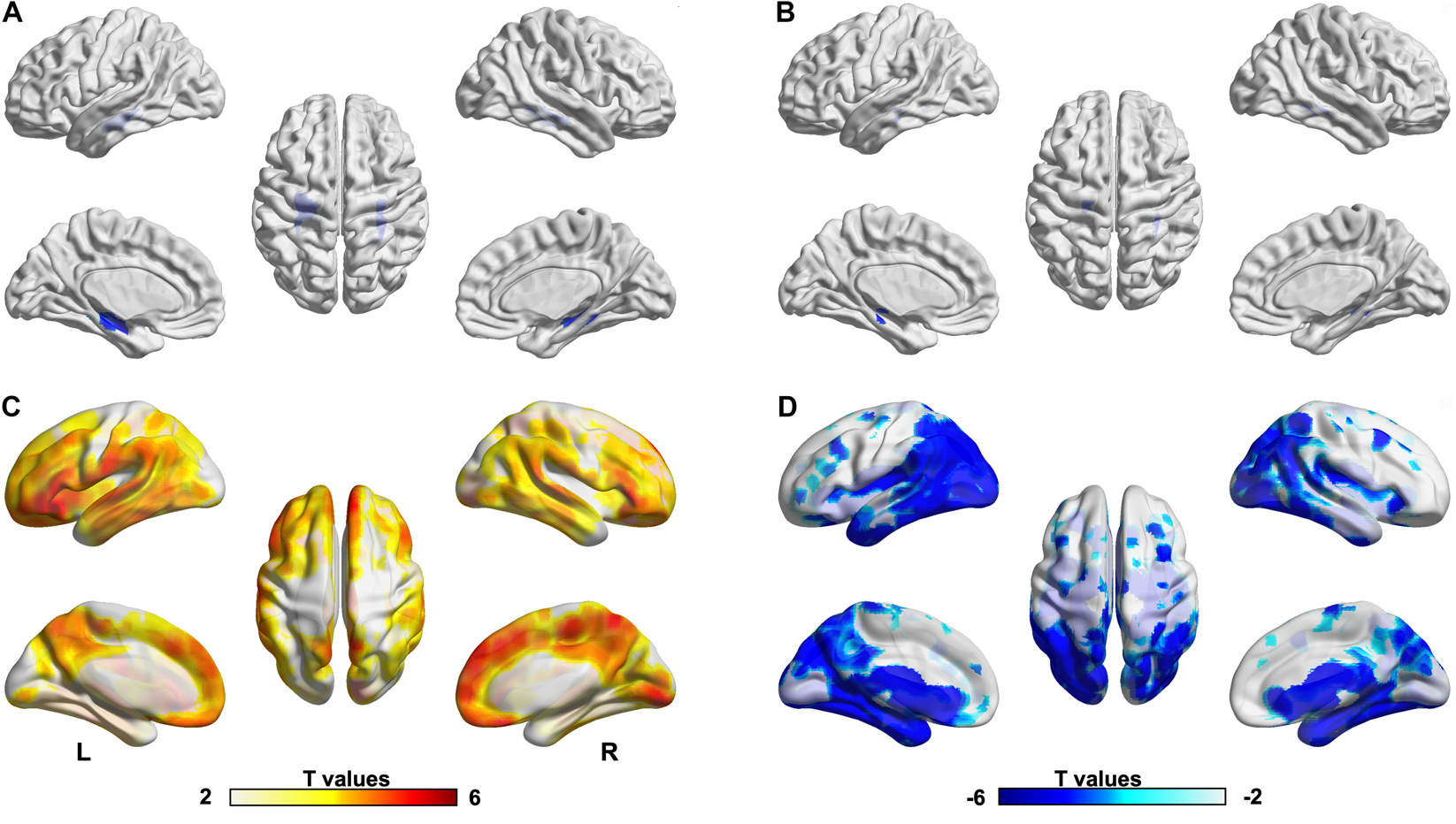


**Supplemental Figure 1. Group difference of mGluR5 expression, amyloid deposition, and glucose metabolism between NC and AD by voxel-wise analysis.**

A. Group difference of mGluR5 availability between NC and AD with pons as the reference and without partial volume correction (PVC), AD patients also show lower mGluR5 availability in the bilateral hippocampus and parahippocampal gyrus than NCs. B. Group difference of mGluR5 availability between NC and AD with cerebellum as the reference after PVC, AD patients have lower mGluR5 availability in the left hippocampus and right posterior cingulate than NCs. C. Group difference of amyloid deposition between NC and AD, AD patients have higher amyloid deposition than NCs in the frontal, parietal, lateral temporal, occipital, precuneus, anterior and posterior cingulate. D. Group difference of glucose metabolism between NC and AD, AD patients have lower glucose metabolism than NCs in the parietal, lateral temporal, medial temporal, occipital lobes, and precuneus.

The color bar in the voxel-wise results represents the T value of the differences among groups with the statistical threshold of *p* < 0.05, peak-level FDR correction, adjusted age, gender, and education years as covariates.


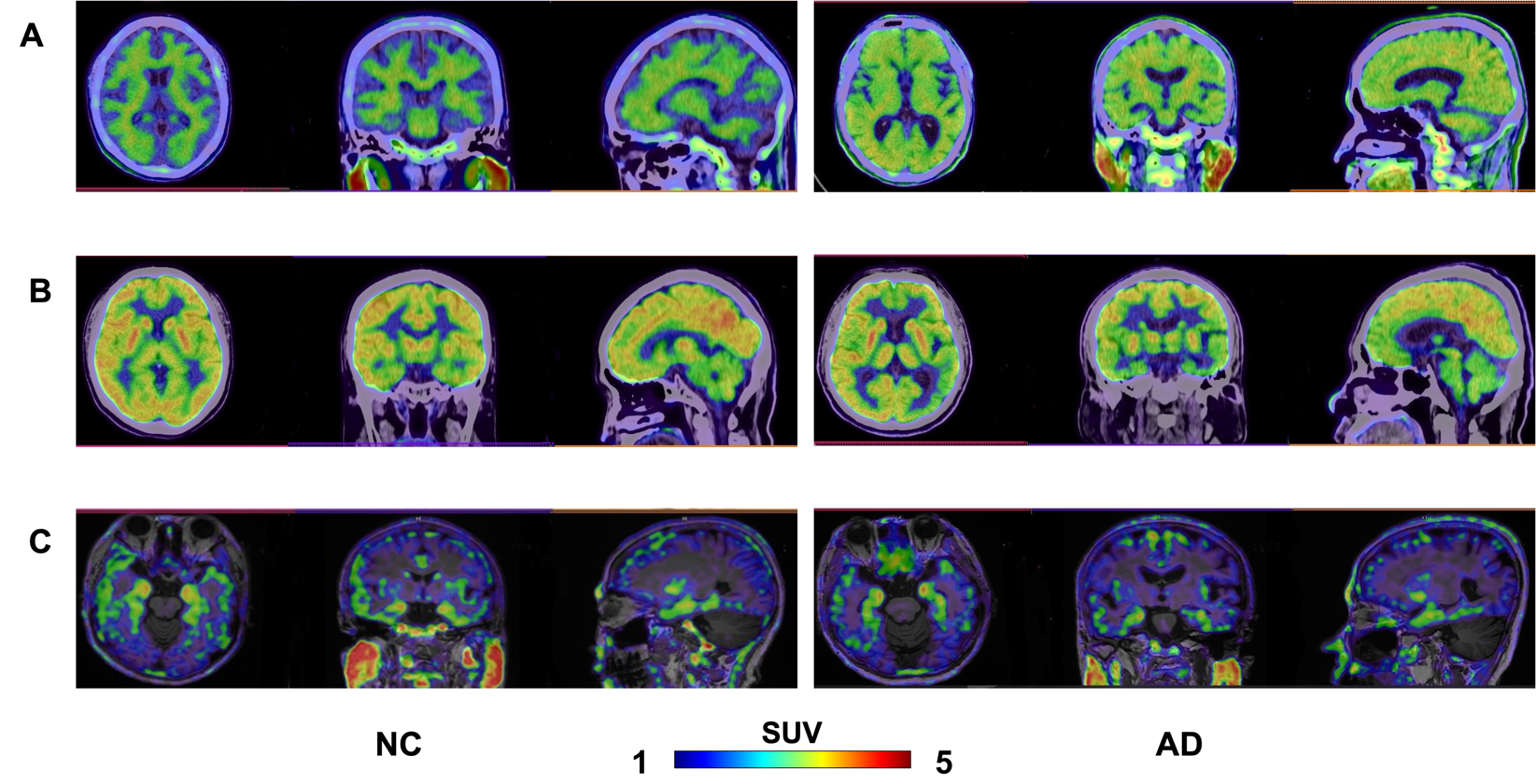


**Supplemental Figure 2**. **Representative PET images in the NC and AD patient from [^18^F]Florbetapir (A), [^18^F]FDG (B), and [^18^F]PSS232 (C).**

A. [^18^F]Florbetapir PET images in the NC and AD individual, the increased uptake of radioactivity in the gray matter of AD was higher than in NC.

B. [^18^F]FDG PET images in the NC and AD individual, compared to the NC, the uptake of radioactivity in the medial temporal, left parietal lobe and lateral temporal lobe were decreased in AD.

C. [^18^F]PSS232 PET images in the NC and AD individual, compared to the NC, the uptake of radioactivity in the bilateral hippocampus and parahippocampal gyrus was decreased in AD.


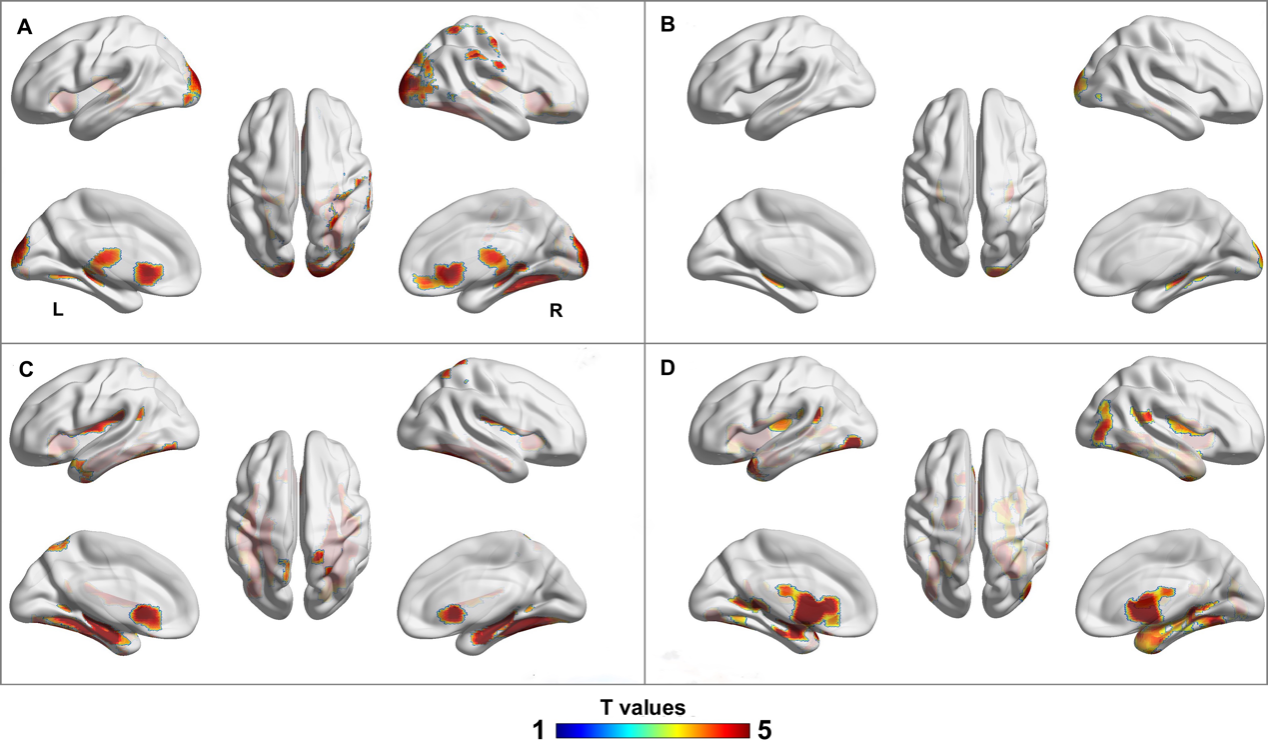


**Supplemental Figure 3. The associations of regional mGluR5 binding with glucose metabolism by voxel-wise analyses in the AD patients and NCs.**

A. In the AD group, the hippocampus [^18^F]PSS232 SUVr was positively associated with glucose metabolism in the bilateral hippocampus, parahippocampal gyrus, insula, superior occipital lobe, and right lateral parietal lobe.

B. In the AD group, the parahippocampal gyrus [^18^F]PSS232 SUVr was positively associated with glucose metabolism in the bilateral hippocampus, right parahippocampal gyrus, and superior occipital lobe.

C. In the NC group, the hippocampus [^18^F]PSS232 SUVr was positively associated with glucose metabolism in the bilateral hippocampus, parahippocampal gyrus, and superior occipital lobe.

D. In the NC group, the parahippocampal gyrus [^18^F]PSS232 SUVr was positively associated with glucose metabolism in the bilateral hippocampus, parahippocampal gyrus, and lateral temporal lobe.

The color bar represents the T value with a statistical threshold of *p* < 0.05, peak-level FDR correction, adjusted age, gender, and education years as covariates.


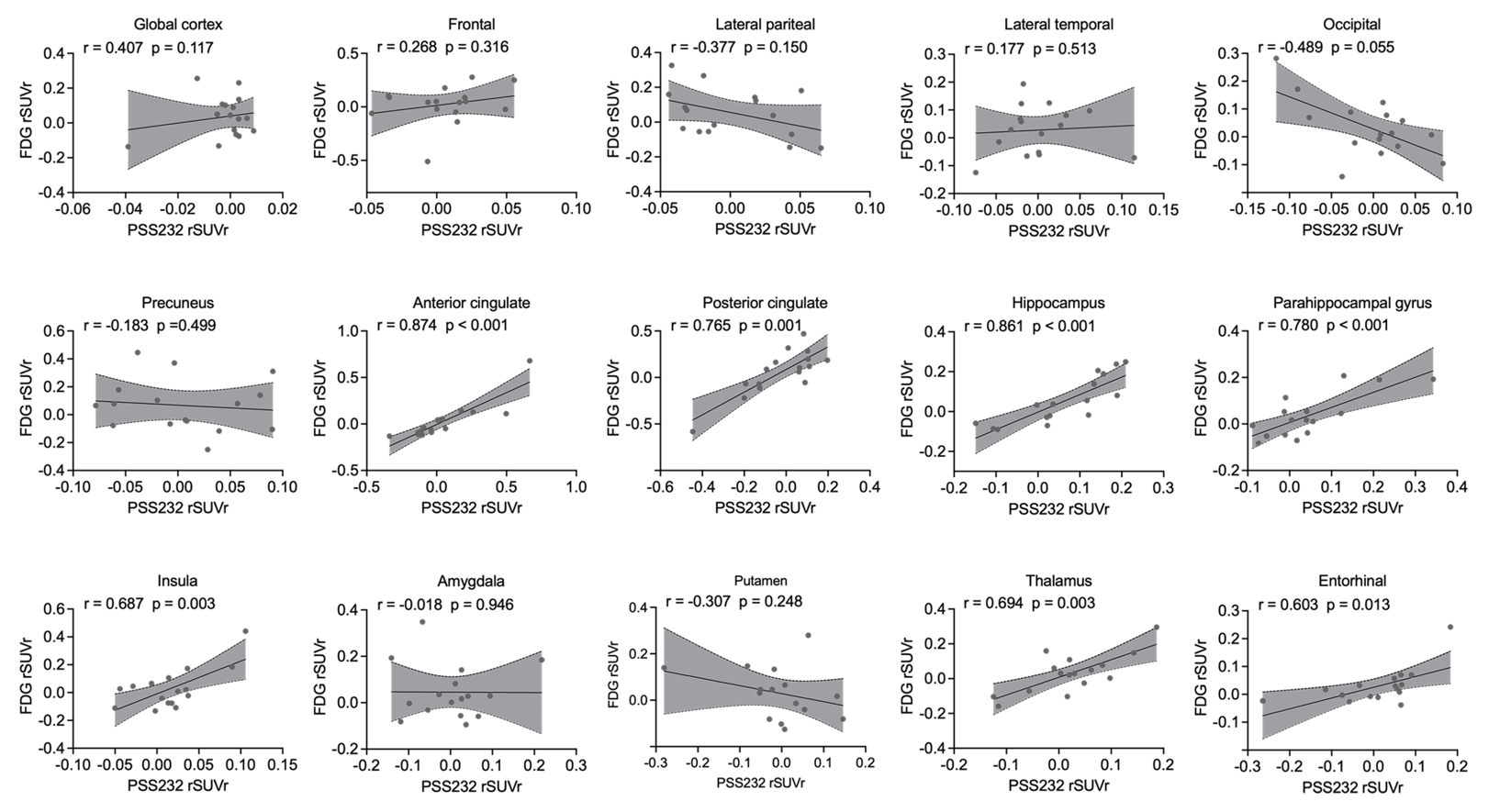


**Supplemental Figure 4. The associations between regional [^18^F]PSS232 SUVr and regional [^18^F]FDG SUVr in the NC group.** The regional [^18^F]PSS232 SUVr was positively associated with regional glucose metabolism in the posterior cingulate cortex, anterior cingulate cortex, hippocampus, parahippocampal gyrus, insula, thalamus, and entorhinal cortex.

The statistical model is partial correlation, with age, education years, and gender as covariates. The dashed lines represent the 95% confidence intervals of the best-fit lines.

**
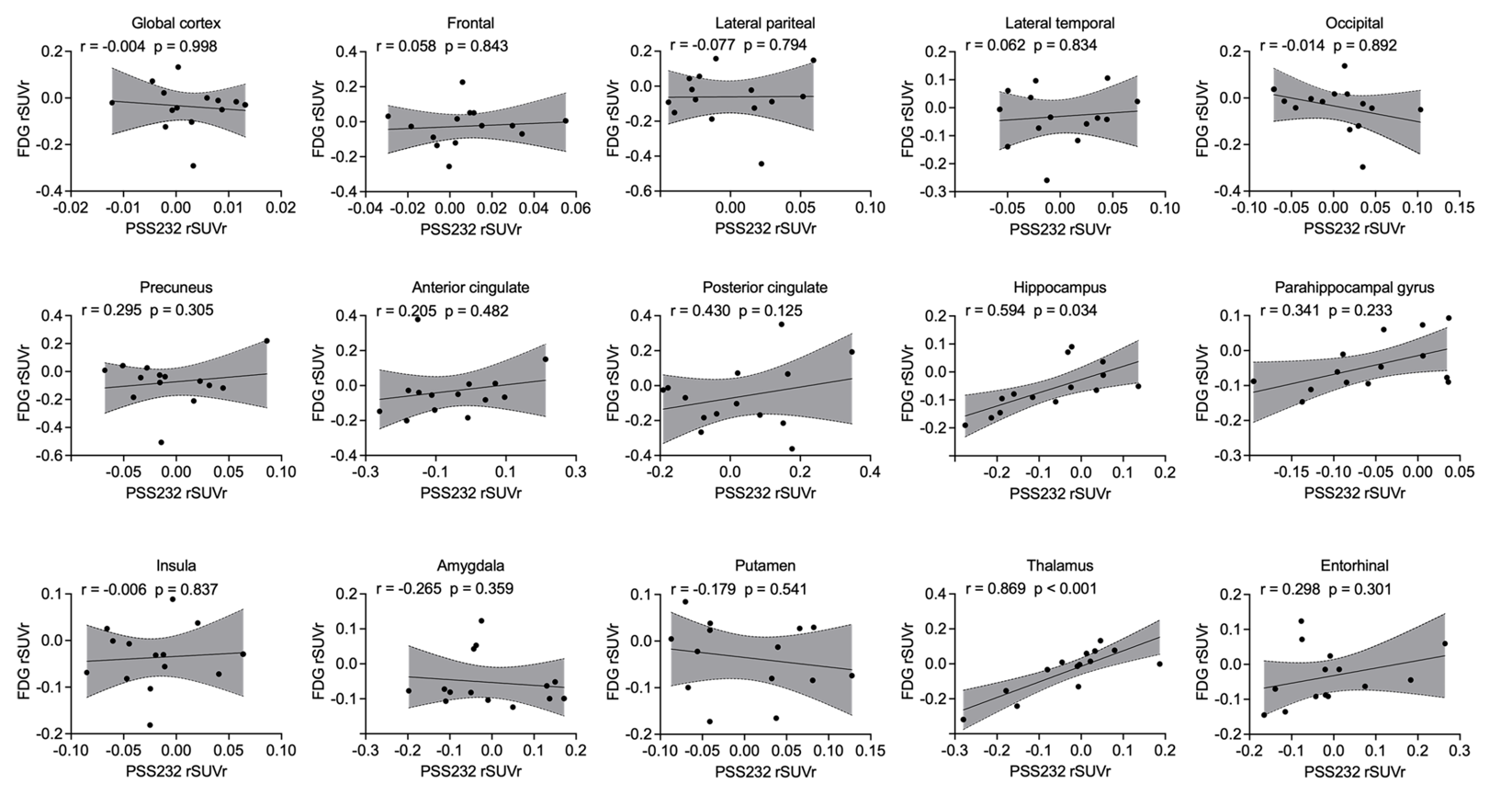
**

**Supplemental Figure 5.** **The associations between regional [^18^F]PSS232 SUVr and regional [^18^F]FDG SUVr in the AD group.** The regional [^18^F]PSS232 SUVr was positively associated with regional glucose metabolism only in the hippocampus and thalamus.

The statistical model is partial correlation, with age, education years, and gender as covariates. The dashed lines represent the 95% confidence intervals of the best-fit lines.
